# Supplementary material for: Host cell CRISPR genomics and modelling reveal shared metabolic vulnerabilities in the intracellular development of Plasmodium falciparum and related hemoparasites
Source: Nat Commun. 2024 Jul 21;15:6145. doi: 10.1038/s41467-024-50405-x (PMC11271486; doi:10.1038/s41467-024-50405-x)
Supplement: Supplementary file 1 — Supplementary Information [file 41467_2024_50405_MOESM1_ESM.pdf]

Supplementary Figures:

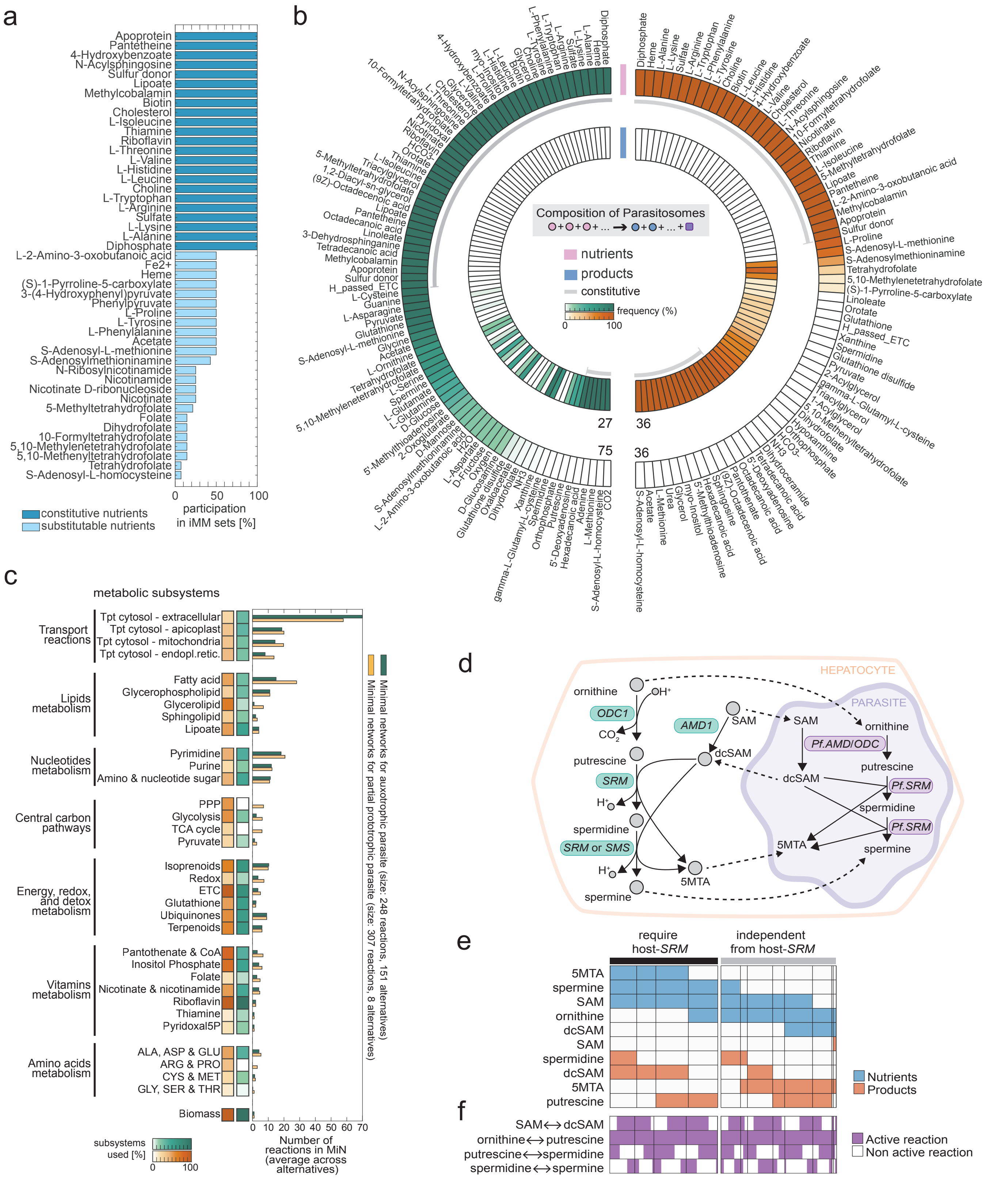

Figure S1. Computational analysis of *P. falciparum* nutritional requirements and dependence on hepatocyte genes. (a) Nutrients that partial prototrophic *P. falciparum* needs to uptake from the hepatocyte to survive. Participation in the alternative iMM sets determines whether each metabolite is constitutive, if it is required in all the alternatives, or substitutable, if another metabolite can substitute its function. **(b)** Metabolic composition of the alternative parasitosomes in case study I (auxotrophic parasite, green) and II (partial prototrophic parasite, orange). The frequency indicates whether the nutrients and products are constitutive if

they appear in all alternative parasitosomes. **(c)** Metabolic subsystems composing the different networks for the parasitosomes, when considering auxotrophic (green) or partial prototrophic (orange) parasites, in terms of number of reactions and percentage of subsystem coverage. **(d)** Host-parasite interactions for polyamine synthesis. **(e)** Nutrients used and products secreted by the auxotrophic parasites into the hepatocyte for the polyamine's pathway. Separated into parasites that require the hepatocyte's *SRM* gene for survival (black) and those that survive independently of the host's *SRM* gene (grey). Column divisions group parasitosomes with the same metabolic flux profile for the polyamine synthesis. **(f)** Activity of the polyamine pathway in parasitosomes in relation to their dependence on the host's *SRM* gene. SAM: adenosylmethionine, dcSAM: decarboxylated SAM, 5MTA: 5-methylthioadenosine.

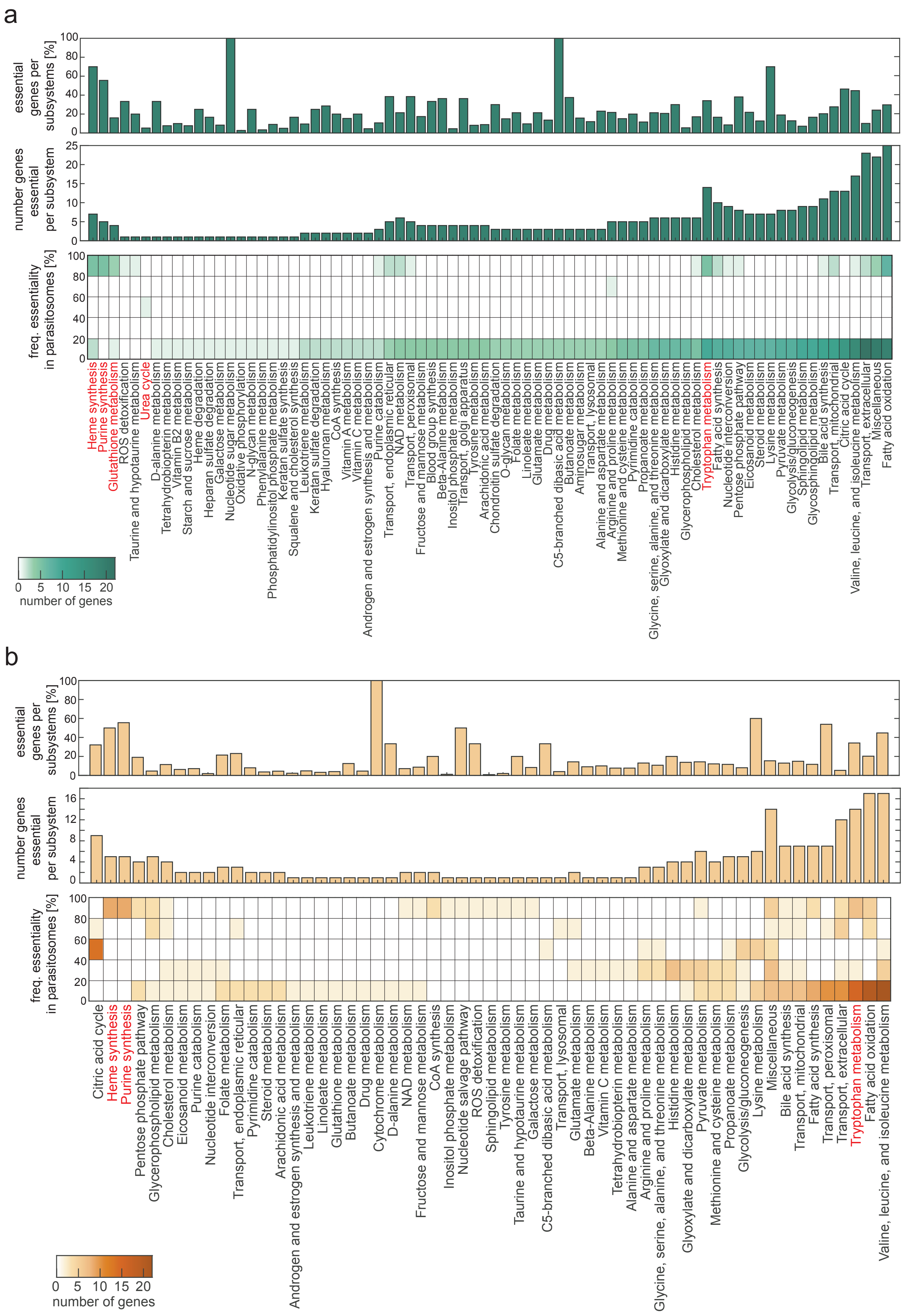

Figure S2. Computational analysis of *P. falciparum* nutritional requirements and dependence on hepatocyte genes. Complete list of metabolic subsystems associated with hepatocyte genes that are

essential for at least one parasitosome. Classification of the genes based on their frequency of essentiality across parasitosomes, the number of essential genes associated with each subsystem and the percentage of essential genes out of the total genes associated with each subsystem in case of auxotrophic parasites **(a)** or partial prototrophic parasites **(b)**.

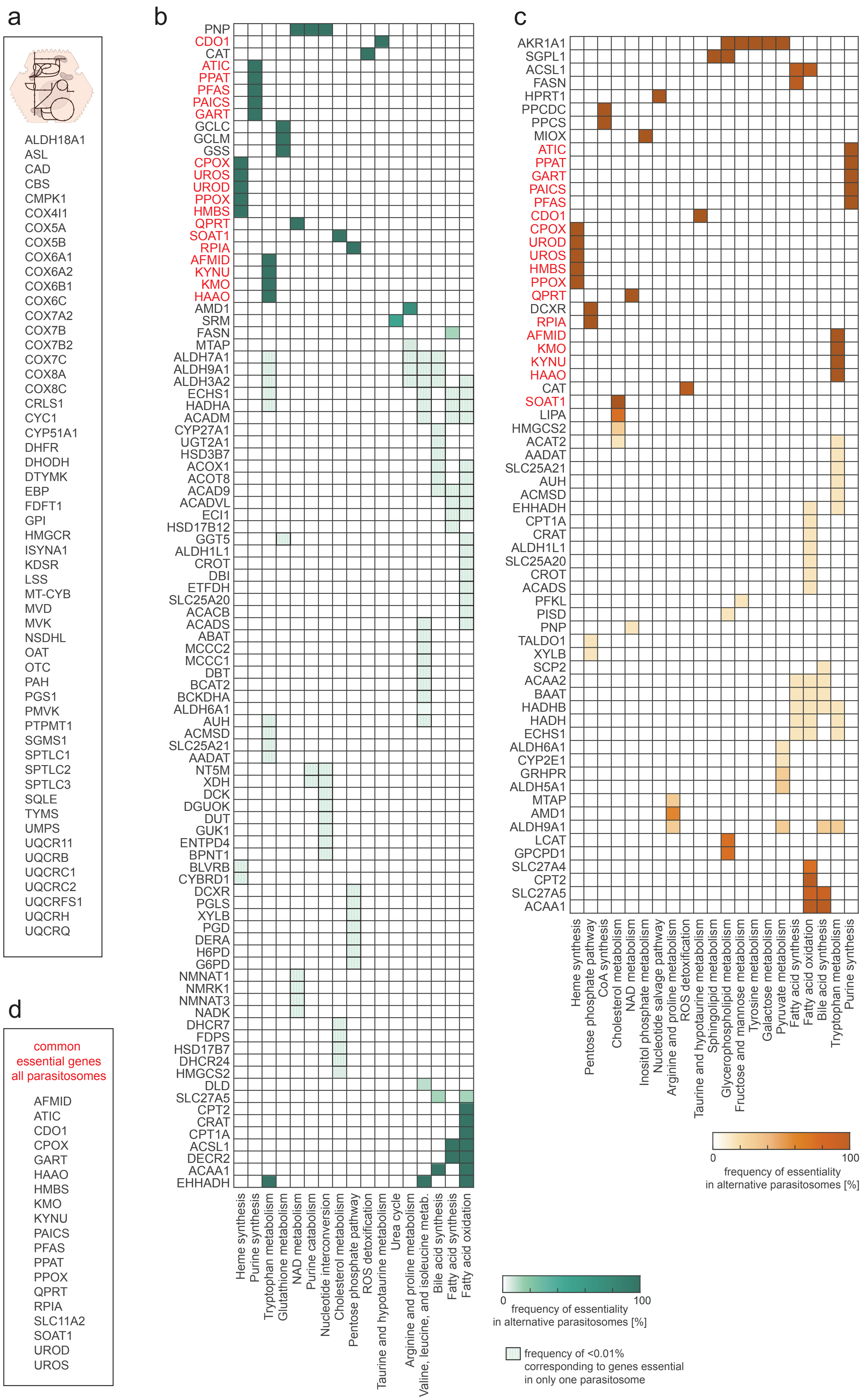
a
ALDH18A1
ASL
CAD
CBS
CMPK1
COX4I1
COX5A
COX5B
COX6A1
COX6A2
COX6B1
COX6C
COX7A2
COX7B
COX7B2
COX7C
COX8A
COX8C
CRLS1
CYC1
CYP51A1
DHFR
DHODH
DTYMK
EBP
FDFT1
GPI
HMGCR
ISYNA1
KDSR
LSS
MT-CYB
MVD
MVK
NSDHL
OAT
OTC
PAH
PGS1
PMVK
PTPMT1
SGMS1
SPTLC1
SPTLC2
SPTLC3
SQLE
TYMS
UMPS
UQCR11
UQCRB
UQCRC1
UQCRC2
UQCRFS1
UQCRH
UQCRQ
b
PNP
CDO1
CAT
ATIC
PPAT
PFAS
PAICS
GART
GCLC
GCLM
GSS
CPOX
UROS
UROD
PPOX
HMBS
QPRT
SOAT1
RPIA
AFMID
KYNU
KMO
HAAO
AMD1
SRM
FASN
MTAP
ALDH7A1
ALDH9A1
ALDH3A2
ECHS1
HADHA
ACADM
CYP27A1
UGT2A1
HSD3B7
ACOX1
ACOT8
ACAD9
ACADVL
ECI1
HSD17B12
GGT5
ALDH1L1
CROT
DBI
ETFDH
SLC25A20
ACACB
ACADS
ABAT
MCCC2
MCCC1
DBT
BCAT2
BCKDHA
ALDH6A1
AUH
ACMSD
SLC25A21
AADAT
NT5M
XDH
DCK
DGUOK
DUT
GUK1
ENTPD4
BPNT1
BLVRB
CYBRD1
DCXR
PGLS
XYLB
PGD
DERA
H6PD
G6PD
NMNAT1
NMRK1
NMNAT3
NADK
DHCR7
FDPS
HSD17B7
DHCR24
HMGCS2
DLD
SLC27A5
CPT2
CRAT
CPT1A
ACSL1
DECR2
ACAA1
EHHADH
Heme synthesis
Purine synthesis
Tryptophan metabolism
Glutathione metabolism
NAD metabolism
Purine catabolism
Nucleotide interconversion
Cholesterol metabolism
Pentose phosphate pathway
ROS detoxification
Taurine and hypotaurine metabolism
Urea cycle
Arginine and proline metabolism
Valine, leucine, and isoleucine metab.
Bile acid synthesis
Fatty acid synthesis
Fatty acid oxidation
0
100
frequency of essentiality
in alternative parasitosomes [%]
frequency of <0.01%
corresponding to genes essential
in only one parasitosome
c
AKR1A1
SGPL1
ACSL1
FASN
HPRT1
PPCDC
PPCS
MIOX
ATIC
PPAT
GART
PAICS
PFAS
CDO1
CPOX
UROD
UROS
HMBS
PPOX
QPRT
DCXR
RPIA
AFMID
KMO
KYNU
HAAO
CAT
SOAT1
LIPA
HMGCS2
ACAT2
AADAT
SLC25A21
AUH
ACMSD
EHHADH
CPT1A
CRAT
ALDH1L1
SLC25A20
CROT
ACADS
PFKL
PISD
PNP
TALDO1
XYLB
SCP2
ACAA2
BAAT
HADHB
HADH
ECHS1
ALDH6A1
CYP2E1
GRHPR
ALDH5A1
MTAP
AMD1
ALDH9A1
LCAT
GPCPD1
SLC27A4
CPT2
SLC27A5
ACAA1
Heme synthesis
Pentose phosphate pathway
CoA synthesis
Cholesterol metabolism
NAD metabolism
Inositol phosphate metabolism
Nucleotide salvage pathway
Arginine and proline metabolism
ROS detoxification
Taurine and hypotaurine metabolism
Sphingolipid metabolism
Glycerophospholipid metabolism
Fructose and mannose metabolism
Tyrosine metabolism
Galactose metabolism
Pyruvate metabolism
Fatty acid synthesis
Fatty acid oxidation
Bile acid synthesis
Tryptophan metabolism
Purine synthesis
0
100
frequency of essentiality
in alternative parasitosomes [%]
d
common
essential genes
all parasitosomes
AFMID
ATIC
CDO1
CPOX
GART
HAAO
HMBS
KMO
KYNU
PAICS
PFAS
PPAT
PPOX
QPRT
RPIA
SLC11A2
SOAT1
UROD
UROS

Figure S3. Hepatocyte genes essential for *P. falciparum* and dispensable for the host. (a) List of genes essential in the healthy hepatocyte model. **(b-c)** Classification of genes per metabolic pathway and the frequency of their essentiality across parasitosomes in the case of auxotrophic (**b**) or partial prototrophic parasites (**c**). Genes essential for all parasitosomes are highlighted in red. **(d)** List of 19 genes essential for all parasitosomes.

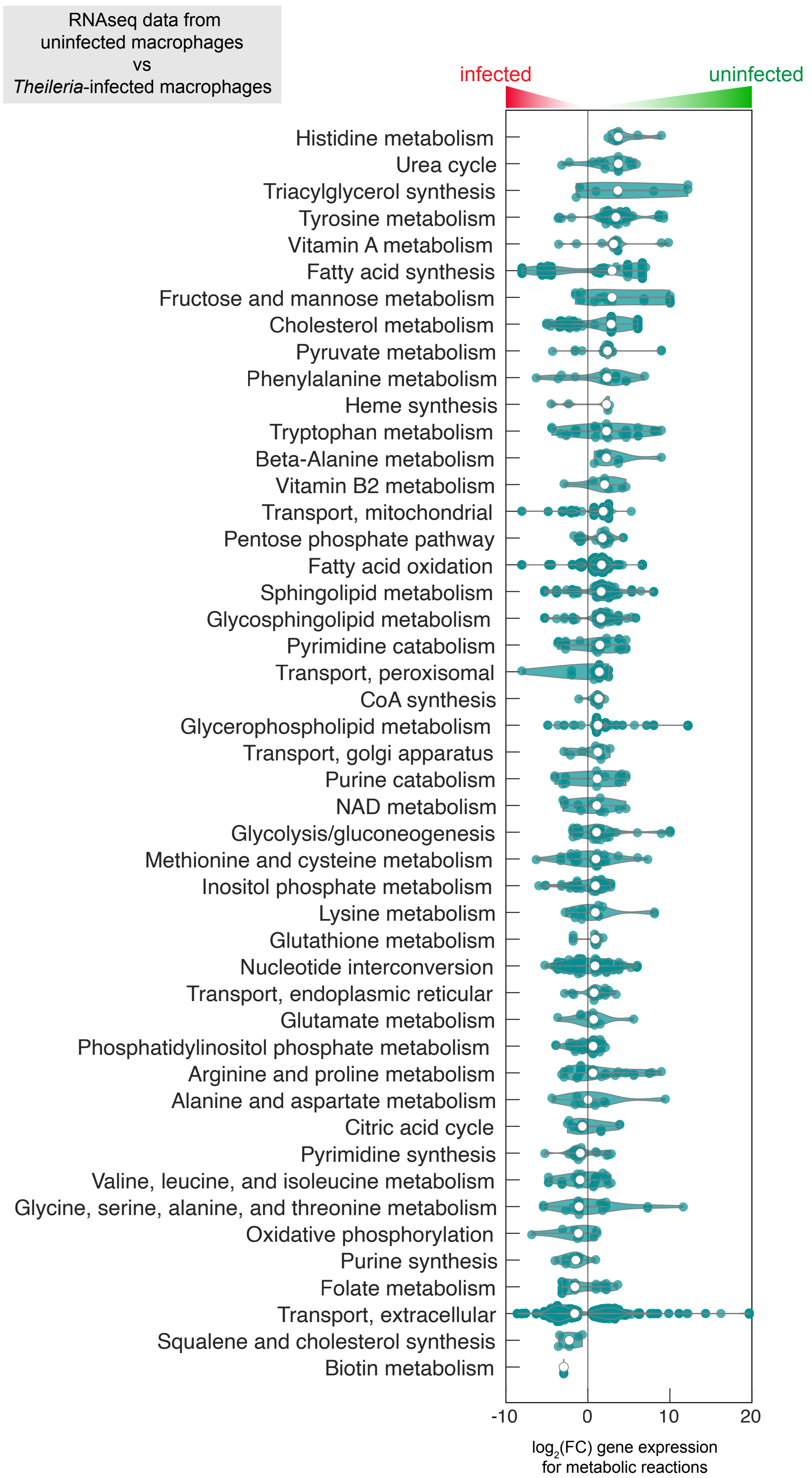

Figure S4. Comparison of host metabolic gene expression in uninfected vs. *Theileria*-infected macrophages. Violin plot of the fold change gene expression deregulation classified in numerous host metabolic pathways in primary bovine macrophages upon *Theileria annulata*-induced transformation.

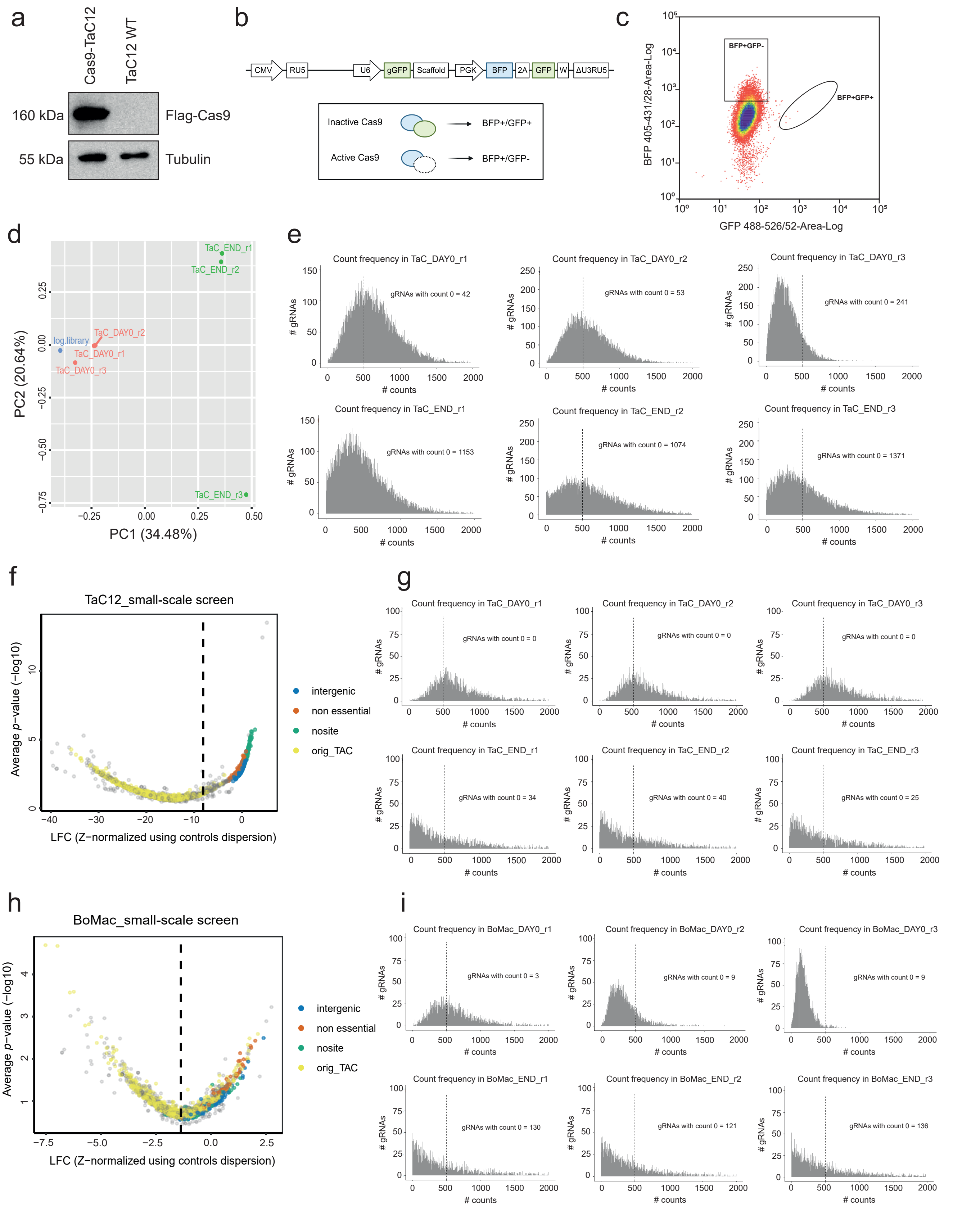

Figure S5. Cas9-expression in *Theileria*-infected macrophages and supplementary data on genetic screens. (a) Western blot of whole cell lysates from Cas9-TaC12 and TaC12 WT. Flag-tagged Cas9 is expressed in TaC12 cells after transduction with Cas9-Flag-Blast lentiviruses. Tubulin staining served as a loading control. **(b)** Schematic representation of the lentiviral vector used for Cas9 activity assay. In transduced cells with no active Cas9 protein, both GFP and BFP fluorescence are detectable. When Cas9 is active, the GFP sequence gets cleaved and only the BFP fluorescence is detected. CMV, CMV promoter; RU5, 5' long terminal repeat; hU6, human U6 promoter; gGFP, sgRNA targeting GFP gene; PGK, mouse

Pgk1 promoter; BFP, blue fluorescent protein gene; 2A, *Thosea asigna* virus 2A peptides; GFP, green fluorescent protein gene; W, Woodchuck hepatitis virus posttranscriptional regulatory element; ΔU3RU5, self-inactivating 3' LTR. **(c)** TaC12-Cas9 expressing cells were analyzed by fluorescence-activated cell sorting (FACS) prior to cell sorting for BFP+/GFP- signal. The cell population expressing an active Cas9 protein (BFP+/GFP-) was sorted twice to enrich for BFP-positive cells. The BFP-positive gate was strictly set to enrich for the brightest fluorescing cells. Most cells transduced with the reporter construct are BFP+/GFP- while a small fraction is BFP/GFP double positive, indicating the absence of active Cas9. The same procedure was performed with BoMac-Cas9 cells. **(d)** Principal component analysis plot of log-norm count distribution of the TaC12 genome-wide screens. DAY0 and END points for each biological replicate are shown, as well as the distribution of the CRISPR library (log.library). **(e)** Library representation at DAY0 and END points of TaC12 genome-wide screen replicates. **(f)** Volcano plot showing the hypergeometric distribution analysis of the CRISPR/Cas9 small-scale screen in TaC12. Each gene is plotted based on its average LFC (z-normalized using the control dispersion) and the negative $\log_{10}$ of its *p*-value. A selected set of 100 genes that were found to be non-essential in TaC12_GW is shown in orange. A group of ~ 800 genes significantly depleted in TaC12_GW (orig_TAC) is shown in yellow. The vertical dotted line corresponds to the average z-normalized LFC of the controls (intergenic and non-targeting) minus 1x standard deviation of the entire genetic screen. The screen was performed in 3 biological replicates. **(g)** Library representation at DAY0 and END points of TaC12 small scale screen replicates. **(h)** Volcano plot showing the hypergeometric distribution analysis of the CRISPR/Cas9 small-scale screen in BoMac. Each gene is plotted based on its average LFC (z-normalized using the control dispersion) and the negative $\log_{10}$ of its p-value. A selected set of 100 genes that were found to be non-essential in TaC12_GW is shown in orange. A group of ~ 800 genes significantly depleted in TaC12_GW (orig_TAC) is shown in yellow. The vertical dotted line corresponds to the average z-normalized LFC of the controls (intergenic and non-targeting) minus 1x standard deviation of the entire genetic screen. The screen was performed in 3 biological replicates. **(i)** Library representation at DAY0 and END points of BoMac small scale screen replicates.

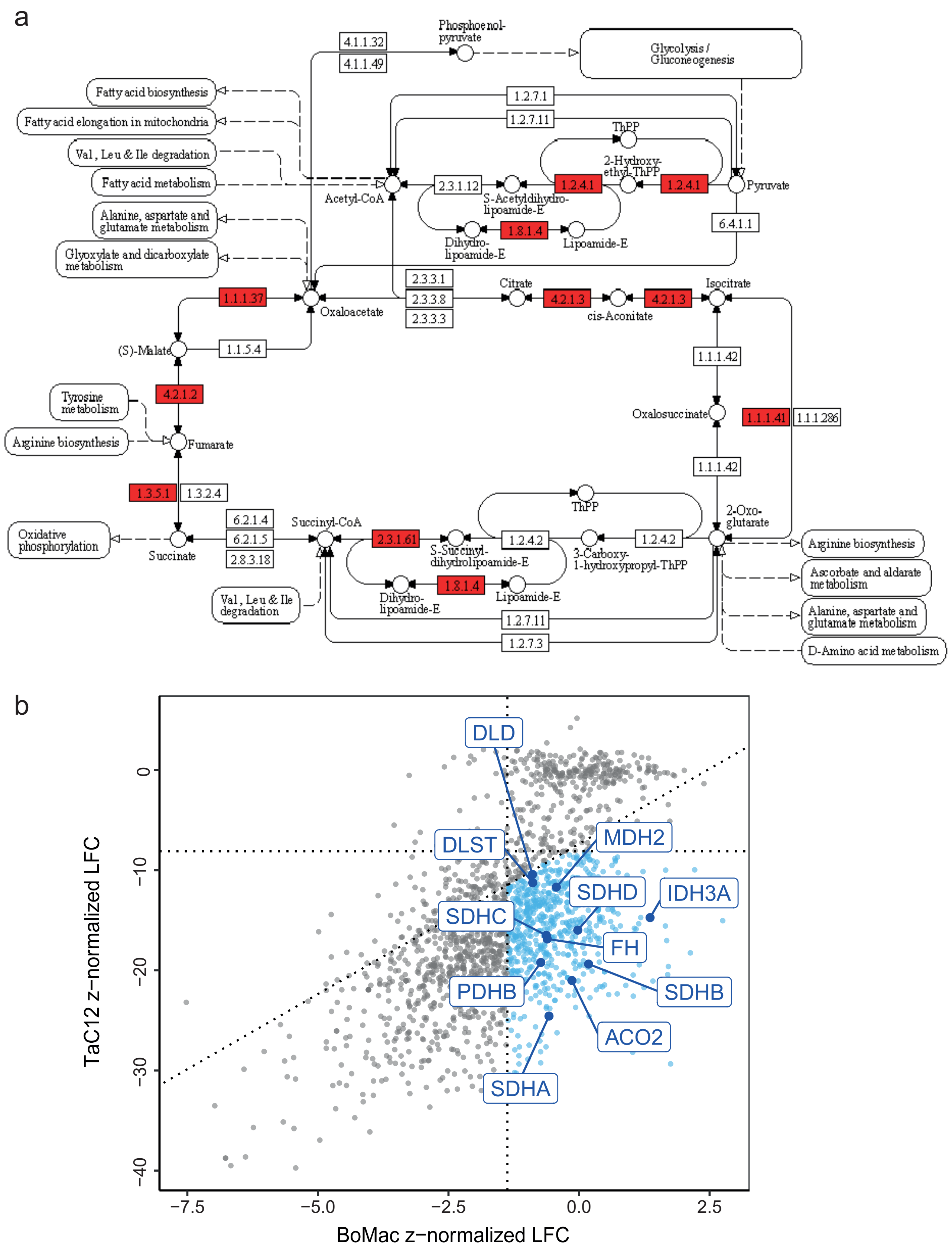

Figure S6. TCA cycle genes are enriched in the *Theileria* essentialome. (a) Representation of the TCA cycle by KEGG. Fitness-conferring genes in TaC12 are shown in red. **(b)** Scoring of genes belonging to the TCA cycle in TaC12 vs BoMac screens.

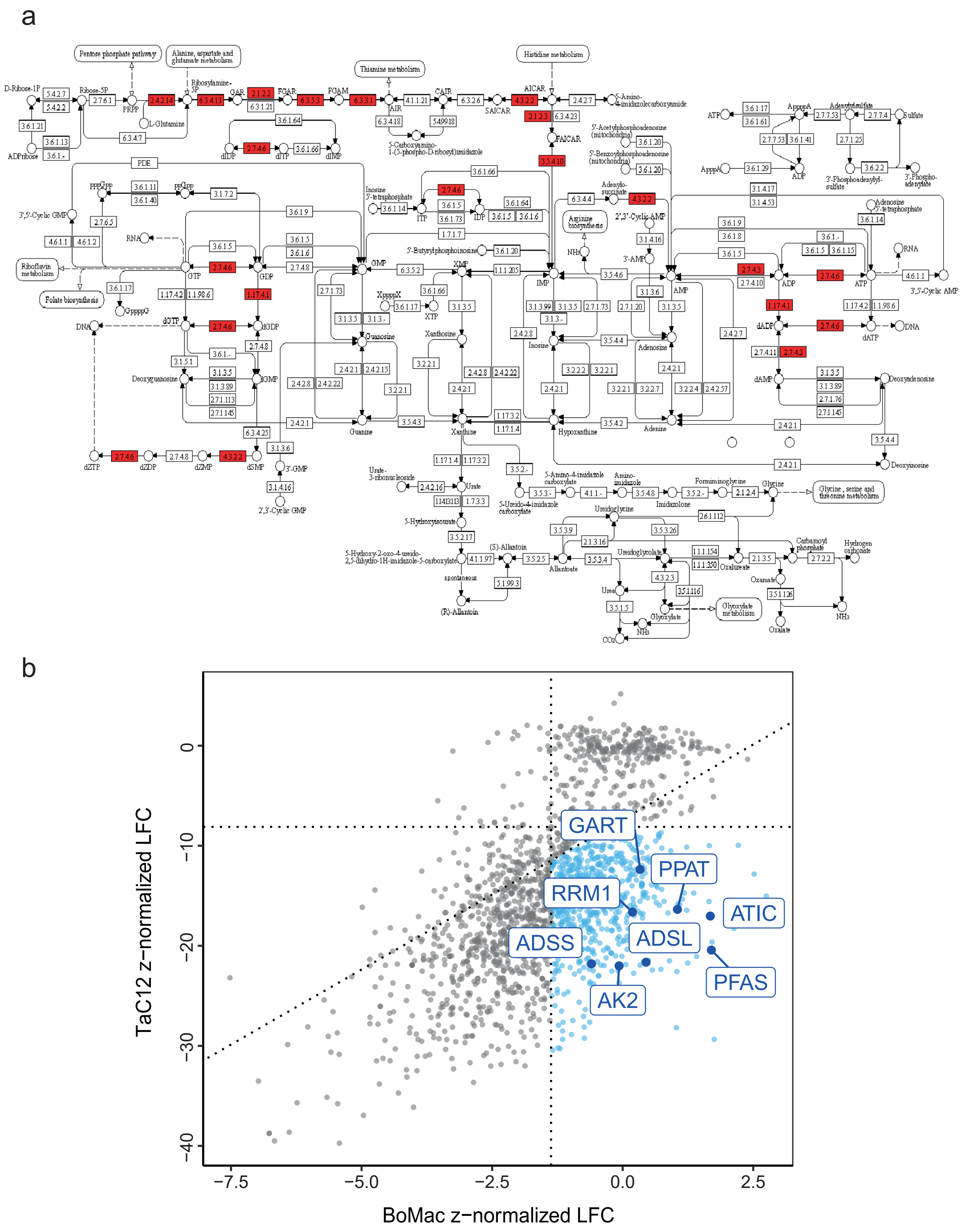

Figure S7. Purine metabolism genes are enriched in the *Theileria* essentialome. (a) Representation of purine metabolism by KEGG. Fitness-conferring genes in TaC12 are shown in red. **(b)** Scoring of genes belonging to the TCA cycle in TaC12 vs BoMac screens.

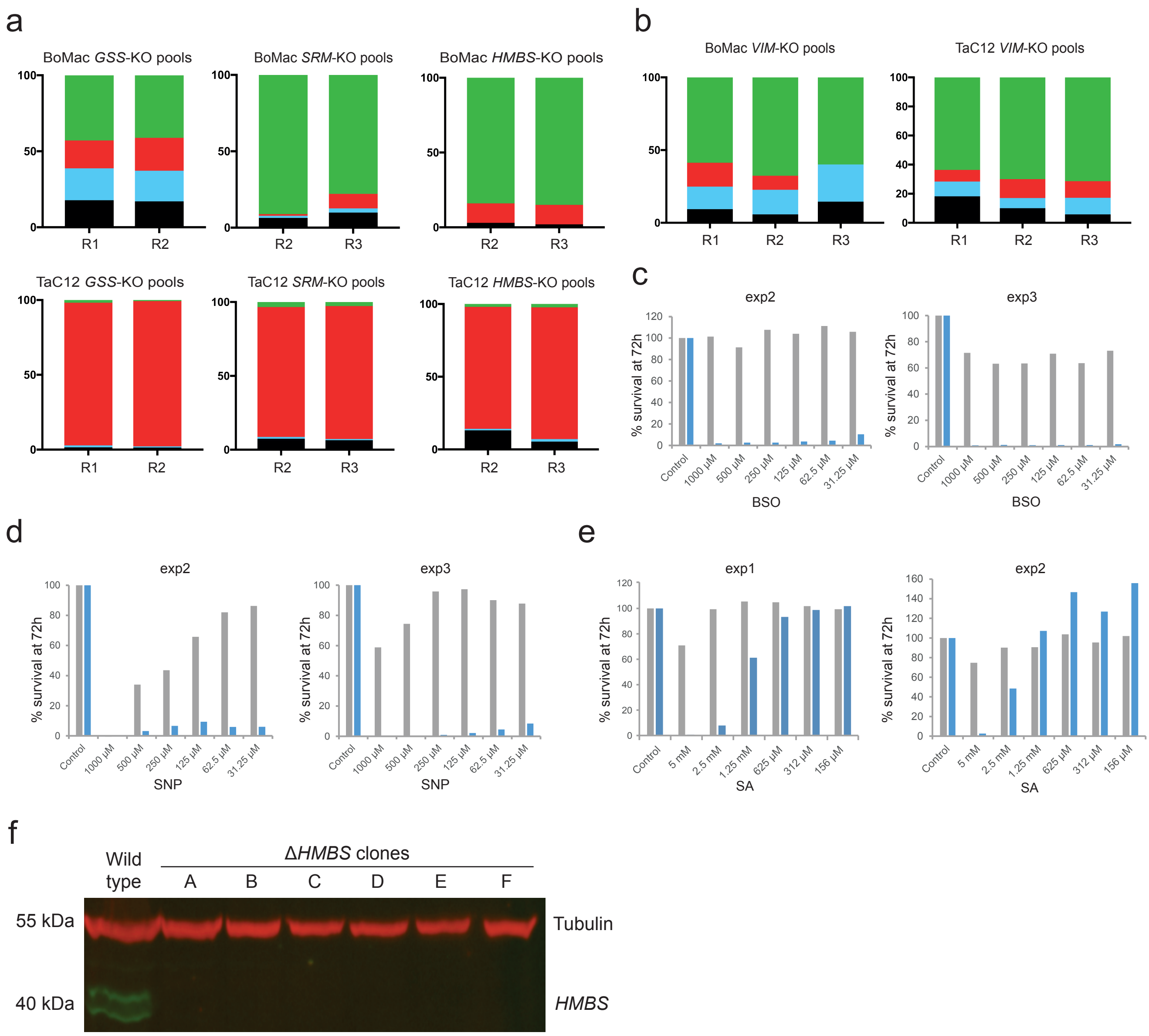

Figure S8. Supplementary data related to Figure 4. (a) TIDE analysis of *GSS*, *SRM* and *HMBS* CRISPR/Cas9 knockouts in TaC12 and BoMac cells (red, WT; green, frameshift mutation; light blue, in-frame mutation; black, not determined) **(b)** TIDE analysis of vimentin (*VIM*) CRISPR/Cas9 knockout in TaC12 and BoMac cells (red, WT; green, frameshift mutation; light blue, in-frame mutation; black, not determined). Vimentin is a non-essential gene in many cell lines, and we use it as a positive control that it can be knocked out in TaC12 and BoMac cells. **(c-e)** Resazurin viability assays showing the percentage of survival of BoMac and TaC12 cells after 72 hours treatment with buthionine sulfoximine (BSO), sodium nitroprusside (SNP), salicylic acid (SA). **(f)** Western blot of whole cell lysates from HAP1 WT and HAP1 Δ*HMBS* clones A-B-C-D-E-F. Tubulin staining served as loading control.

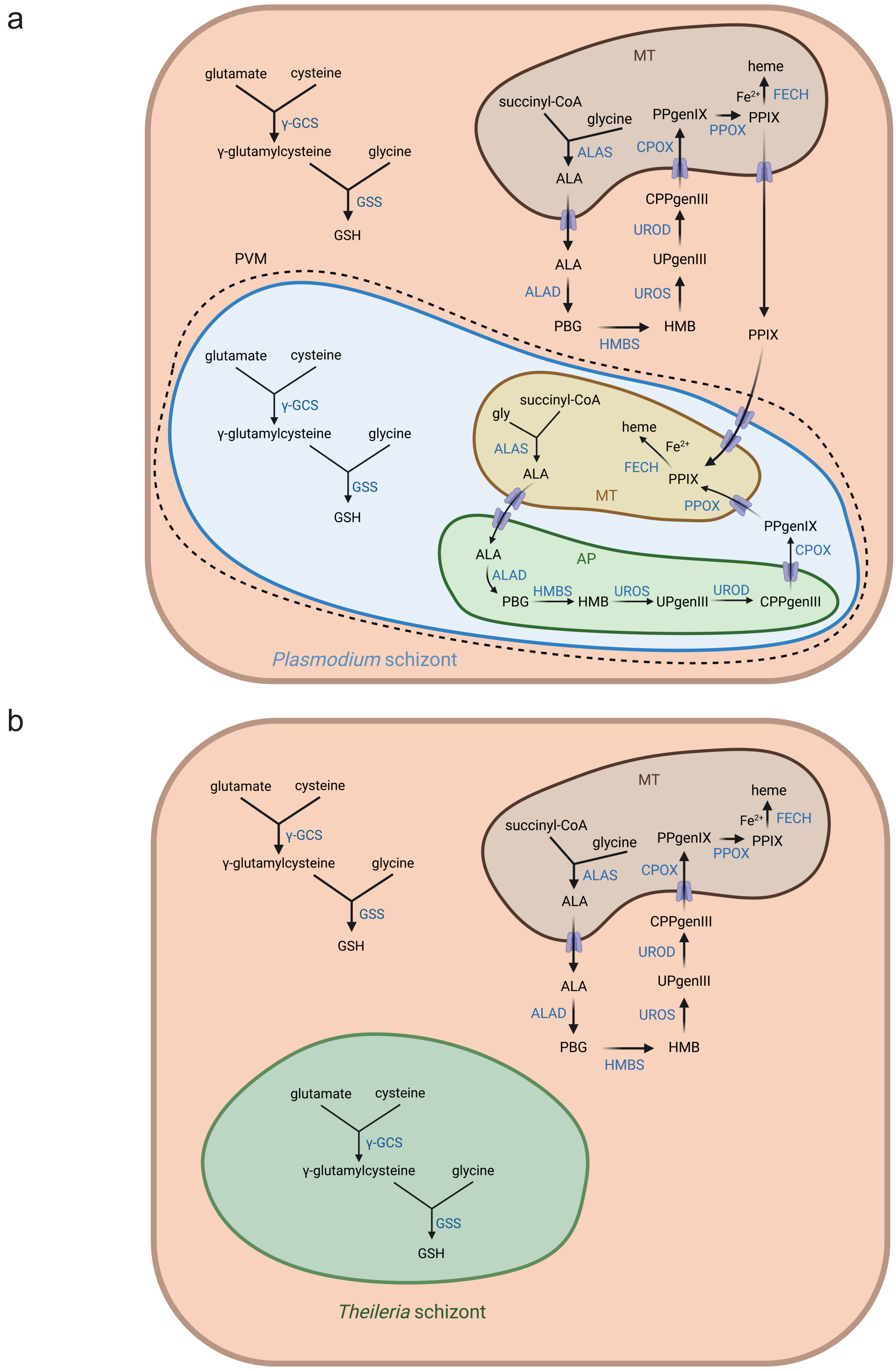

Figure S9. Representation of metabolic pathways for heme and glutathione biosynthesis in parasite-infected host cell. (a) *Plasmodium* parasites have the ability to synthesize both heme and glutathione endogenously. **(b)** *Theileria* parasites can synthesize glutathione but do not encode for heme biosynthetic enzymes.

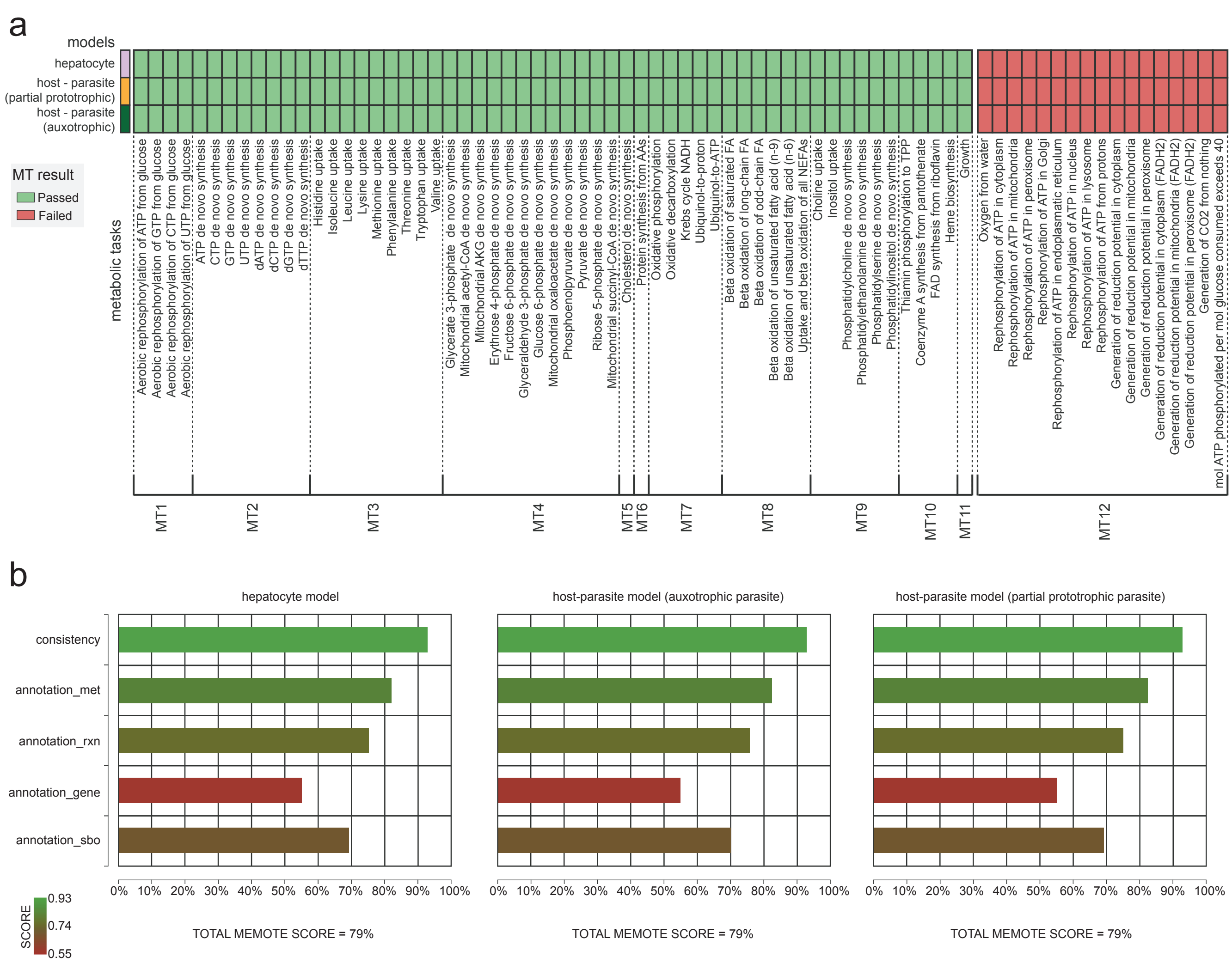

Figure S10. Quality evaluation of model reconstructions. (a) Human metabolic tasks (MTs) captured by the host, and host-parasite metabolic models (green) and those not captured by the models (red). MT1: rephosphorylation of nucleoside triphosphates, MT2: de novo synthesis of nucleotides, MT3: uptake of essential amino acids, MT4: de novo synthesis of key intermediates, MT5: de novo synthesis of other compounds, MT6: protein turnover, MT7: electron transport chain and TCA, MT8: beta-oxidation of fatty acids, MT9: de novo synthesis of phospholipids, MT10: vitamins and co-factors, MT11: growth. M12: leakage tasks. **(b)** MEMOTE score for the host and host-parasite metabolic models.

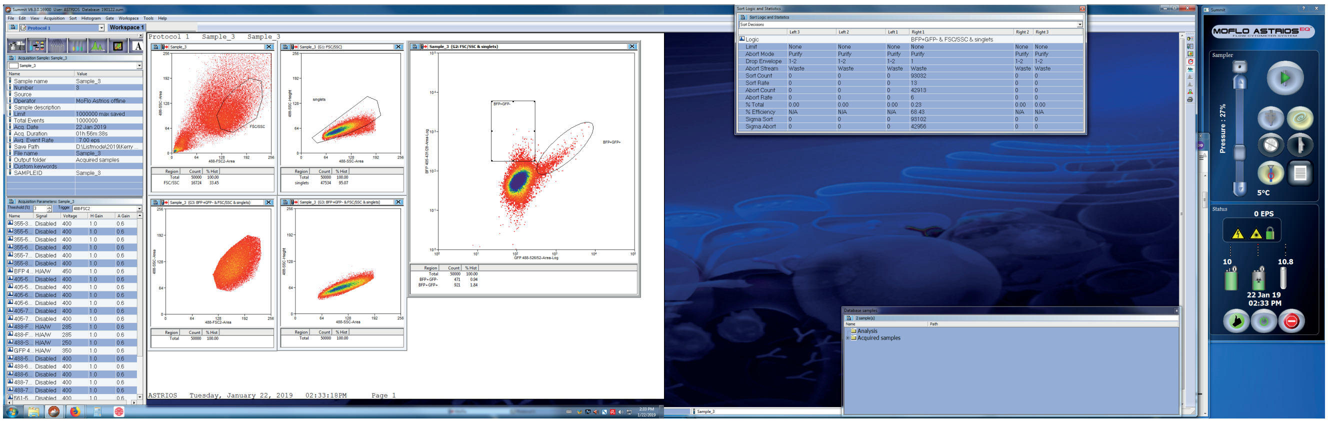

Figure S11. Gating strategy to sort GFP-ve (Cas9 active) *Theileria*-infected macrophages. TaC12 cells were transduced to express pKLV2-U6gRNA5(gGFP)-PGKBFP2AGFP-W reporter plasmid. Side scatter (SSC) versus forward scatter (FCS) was plotted to gate for live cells. Area versus height of SSC was plotted to gate for “singlets”. BFP versus GFP signal was plotted to gate for BFP+/GFP- cells (Cas9 active).

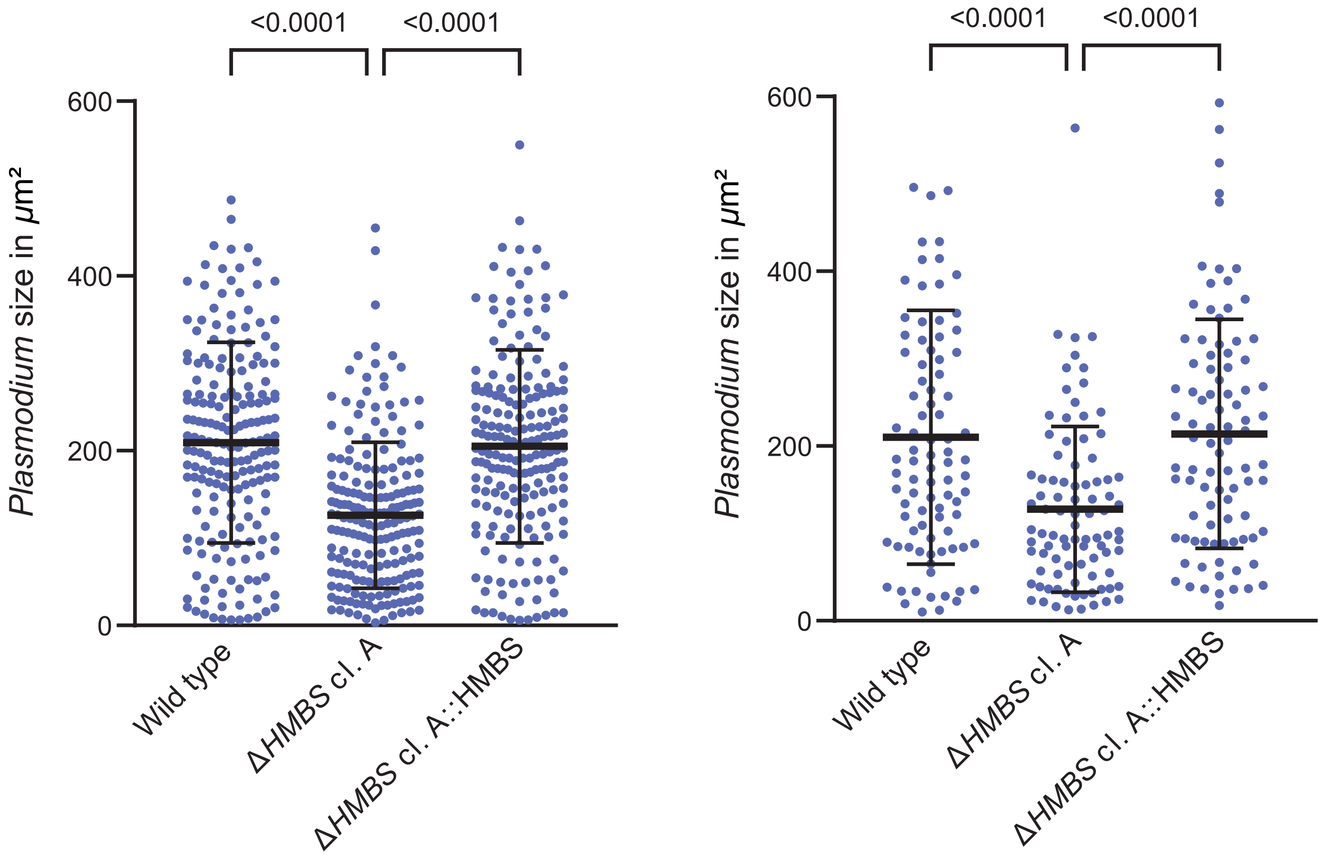

Figure S12. Supplementary datasets of parasite size. Analysis at 48 hpi in HAP1 WT, Δ*HMBS* cl. A, and Δ*HMBS* cl. A::HMBS complementation: 2 additional biological replicates are shown. The graph includes Median parasites size (n=90 and 95) with interquartile range and *p*-values of one-way ANOVA analysis with Dunnett’s multiple comparison test.

Supplementary Tables:

Table S1. Plasmids employed in the study.

Purpose	Plasmid name	Source
Lentivirus production	psPAX2 packaging plasmid	Addgene, #12260
	pMD2-G VSV-G envelope plasmid	Addgene, #12259
Generation of Cas9-expressing cell lines	Cas9-Flag-Blast pXPR_BRD107	Broad Institute
Fluorescence-based reporter assay for selection of Cas9-expressing cells	pKLV2-U6gRNA5(gGFP)-PGKBFP2AGFP-W reporter plasmid	Addgene, #67980
Generation of HAP1 Δ*HMBS* clones	pCRISPR_v3.1	Modified from Addgene, #62988
Re-expression of HMBS in HAP1 Δ*HMBS* clone A and single CRISPR/Cas9 KO of bovine candidate genes	pCMV-puroR	Modified from pcDNA4/TO (Invitrogen)

Table S2. sgRNAs designed for single CRISPR/Cas9 knockout of candidate genes.

Target gene	sgRNA name	sgRNA sequence (5' – 3')
Human *HMBS*	h*HMBS*g1	GCAGCTTGCTCGCATACAGA
Bovine *GSS*	b*GSS*g1	GACTCACCGATGCACAGCTG
Bovine *SRM*	b*SRM*g1	GGACGGCGTTATCCAGTGCA
Bovine *HMBS*	b*HMBS*g1	TCAAGAATCTTGTCCCCCG
Bovine *VIM*	b*VIM*g3	AACGACAAAGCCCGCGTCG

Table S3. Sequences of primers. Lower-case letters indicate overhangs designed for cloning with NEBuilder HiFi DNA Assembly Master Mix.

Purpose	Sequence (5' – 3')	Orientation
TIDE analysis for b*GSS*g1	AGAAGAATGATTCCACTGGG	Forward
	ACTAAAGTCCAGCCTTCAAA	Reverse
TIDE analysis for b*SRM*g1	GCATCCTTCTCGCTGGGCT	Forward
	CAGACACAGCTCCCTGGAAC	Reverse
TIDE analysis for b*HMBS*g1	TTCTGGACAGGAATGGAAGC	Forward
	AGATCCAGGACTCTCTCCTT	Reverse
TIDE analysis for b*VIM*g3	CAACACCGAGTTCAAGAACA	Forward
	TTTCTCAAAGTTTGGGGAAA	Reverse
TIDE analysis for h*HMBS*g1 and screening of HAP1 Δ*HMBS* clones	CAGAGGGTTAGTTCCTAGTA	Forward
	AGAGAGTGCAGTATCAAGAA	Reverse
Amplification of HMBS_232 from cDNA for cloning in pCMV-puroR	actctagaggatccgccaccATGTCTGGTAACGGCAATGC	Forward
	gtcctggttcgtgtggacctGTGATGGTGGTGATGATGATG	Reverse
Amplification of pCMV-puroR as backbone plasmid	GGTGGCGGATCCTCTAGAGT	Forward
	AGGTCCACACGAACCAGGAC	Reverse
