# Supplementary material for: Host cell CRISPR genomics and modelling reveal shared metabolic vulnerabilities in the intracellular development of Plasmodium falciparum and related hemoparasites
Source: Nat Commun. 2024 Jul 21;15:6145. doi: 10.1038/s41467-024-50405-x (PMC11271486; doi:10.1038/s41467-024-50405-x)
Supplement: Supplementary file 3 — Description of Additional Supplementary Files [file 41467_2024_50405_MOESM3_ESM.docx]

**Description of additional supplementary files**

**Supplementary data 1.**

**Title:** Nutrients for different metabolic configurations of *P. falciparum*.

**Description:** List of nutrients used by *Plasmodium* *falciparum* in the liver-iPfa model, when we simulate an auxotrophic parasite (165 nutrients) or a partially prototrophic parasite (47 nutrients). THF: tetrahydrofolate.

**Supplementary data 2.**

**Title:** *in silico* gene essentiality predicted for *P. falciparum* in the liver stage.

**Description:** List of genes resulting essential for parasite’s survival when growing inside the hepatocyte in an auxotrophic mode (209 genes) or in a partial prototrophic mode (110 genes). Frequency of essentiality for each gene across parasitosomes within each case. A frequency of 100%, means the gene is essential for all parasitosomes in the corresponding case.

**Supplementary data 3.**

**Title**: Hypergeometric analysis of bovine genome-wide and small-scale CRISPR screens in TaC12 and BoMac cells.

**Description:** Average LFC values of the three screening replicates (Average_LFC; i_Average_LFC; u_Average_LFC) were normalized using the dispersion of intergenic and non-target controls (z-norm LFC; i_z_LFC; u_z_LFC).

**Supplementary data 4.**

**Title:** Fitness-scoring genes in small-scale CRISPR screen in TaC12 cells and dispensable for BoMac (*Theileria* essentialome).

**Description:** Average LFC values of the three screening replicates (i_Average_LFC; u_Average_LFC) were normalized using the dispersion of intergenic and non-target controls (i_z_LFC; u_z_LFC). Gene Ontology analysis of *Theileria* essentialome (ShinyGO v.066)

**Supplementary data 5.**

**Title:** Genome wide bovine library.

**Description:** Chip files for the genome wide bovine library (CP1273, 85155 distinct barcodes).

**Supplementary data 6.**

**Title.** Bovine sublibrary.

**Description:** Chip file for the bovine sublibrary (CP1504, 17596 distinct barcodes).
