# Supplementary material for: Host cell CRISPR genomics and modelling reveal shared metabolic vulnerabilities in the intracellular development of Plasmodium falciparum and related hemoparasites
Source: Nat Commun. 2024 Jul 21;15:6145. doi: 10.1038/s41467-024-50405-x (PMC11271486; doi:10.1038/s41467-024-50405-x)
Supplement: Supplementary file 10 — Reporting Summary [file 41467_2024_50405_MOESM10_ESM.pdf]

# nature portfolio

Corresponding author(s): Philipp Olias / Sven Rottenberg

Last updated by author(s): 05/30/2024



# Reporting Summary

Nature Portfolio wishes to improve the reproducibility of the work that we publish. This form provides structure for consistency and transparency in reporting. For further information on Nature Portfolio policies, see our Editorial Policies and the Editorial Policy Checklist.

## Statistics

For all statistical analyses, confirm that the following items are present in the figure legend, table legend, main text, or Methods section.

| n/a | Confirmed | |
|---|---|---|
| ☐ | ☒ | The exact sample size (*n*) for each experimental group/condition, given as a discrete number and unit of measurement |
| ☐ | ☒ | A statement on whether measurements were taken from distinct samples or whether the same sample was measured repeatedly |
| ☐ | ☒ | The statistical test(s) used AND whether they are one- or two-sided<br>*Only common tests should be described solely by name; describe more complex techniques in the Methods section.* |
| ☐ | ☒ | A description of all covariates tested |
| ☐ | ☒ | A description of any assumptions or corrections, such as tests of normality and adjustment for multiple comparisons |
| ☐ | ☒ | A full description of the statistical parameters including central tendency (e.g. means) or other basic estimates (e.g. regression coefficient) AND variation (e.g. standard deviation) or associated estimates of uncertainty (e.g. confidence intervals) |
| ☐ | ☒ | For null hypothesis testing, the test statistic (e.g. *F*, *t*, *r*) with confidence intervals, effect sizes, degrees of freedom and *P* value noted<br>*Give P values as exact values whenever suitable.* |
| ☒ | ☐ | For Bayesian analysis, information on the choice of priors and Markov chain Monte Carlo settings |
| ☒ | ☐ | For hierarchical and complex designs, identification of the appropriate level for tests and full reporting of outcomes |
| ☒ | ☐ | Estimates of effect sizes (e.g. Cohen's *d*, Pearson's *r*), indicating how they were calculated |

*Our web collection on statistics for biologists contains articles on many of the points above.*

## Software and code

Policy information about availability of computer code

| | |
|---|---|
| Data collection | Custom code was written in MATLAB 2021b and is available on the following GitHub repository: https://github.com/EPFL-LCSB/host_parasite_interactions. Data for model reconstruction was obtained from public sources, for which the references are provided in the main text and a copy has been included in the data/ folder of the repository. |
| Data analysis | Data analysis was done with MATLAB 2021b and CPLEX 12.10 and the results are available on the corresponding GitHub repository |

For manuscripts utilizing custom algorithms or software that are central to the research but not yet described in published literature, software must be made available to editors and reviewers. We strongly encourage code deposition in a community repository (e.g. GitHub). See the Nature Portfolio guidelines for submitting code & software for further information.

## Data

Policy information about availability of data

All manuscripts must include a data availability statement. This statement should provide the following information, where applicable:

- Accession codes, unique identifiers, or web links for publicly available datasets
- A description of any restrictions on data availability
- For clinical datasets or third party data, please ensure that the statement adheres to our policy

The data and models to generate the results for the hepatocyte - P. falciparum metabolic interactions for this paper are available under the APACHE 2.0 license at https://github.com/EPFL-LCSB/host_parasite_interactions.

CRISPR screen data for this study have been deposited in the European Nucleotide Archive (ENA) at EMBL-EBI under accession number PRJEB73635 and accession numbers for the RNA-seq data for uninfected macrophages (ERS16273013, ERS16273014, ERS16273015) and TaC12 cells (ERS18408949, ERS18408950, ERS18408951) (https://www.ebi.ac.uk/ena/).

## Research involving human participants, their data, or biological material

Policy information about studies with human participants or human data. See also policy information about sex, gender (identity/presentation), and sexual orientation and race, ethnicity and racism.

| | |
|---|---|
| Reporting on sex and gender | Research in this study does not involve human participants, their data, or biological material. |
| Reporting on race, ethnicity, or other socially relevant groupings | Research in this study does not involve human participants, their data, or biological material. |
| Population characteristics | Research in this study does not involve human participants, their data, or biological material. |
| Recruitment | Research in this study does not involve human participants, their data, or biological material. |
| Ethics oversight | Research in this study does not involve human participants, their data, or biological material. |

Note that full information on the approval of the study protocol must also be provided in the manuscript.

# Field-specific reporting

Please select the one below that is the best fit for your research. If you are not sure, read the appropriate sections before making your selection.

[x] Life sciences [ ] Behavioural & social sciences [ ] Ecological, evolutionary & environmental sciences

For a reference copy of the document with all sections, see nature.com/documents/nr-reporting-summary-flat.pdf

# Life sciences study design

All studies must disclose on these points even when the disclosure is negative.

| | |
|---|---|
| Sample size | No sample size calculation was performed as this study does not involve in vivo experiments or sampling of a larger population. |
| Data exclusions | No data was excluded. |
| Replication | Attempts of replication were successful. |
| Randomization | No experimental groups (e.g. mice) were compared in this study. |
| Blinding | Blinding was not required as no experimental groups were compared. |

# Reporting for specific materials, systems and methods

We require information from authors about some types of materials, experimental systems and methods used in many studies. Here, indicate whether each material, system or method listed is relevant to your study. If you are not sure if a list item applies to your research, read the appropriate section before selecting a response.

### Materials & experimental systems

| n/a | Involved in the study | |
|---|---|---|
| [ ] | [x] | Antibodies |
| [ ] | [x] | Eukaryotic cell lines |
| [x] | [ ] | Palaeontology and archaeology |
| [ ] | [x] | Animals and other organisms |
| [x] | [ ] | Clinical data |
| [x] | [ ] | Dual use research of concern |
| [x] | [ ] | Plants |

### Methods

| n/a | Involved in the study | |
|---|---|---|
| [x] | [ ] | ChIP-seq |
| [x] | [ ] | Flow cytometry |
| [x] | [ ] | MRI-based neuroimaging |

## Antibodies

| | |
|---|---|
| Antibodies used | rabbit anti-HMBS (Sigma HPA050659, dilution 1:1000)<br>mouse anti-tubulin (Sigma T9026, dilution 1:5000) |

| | |
|---|---|
| | mouse anti-Flag M2 (Sigma F3165, dilution 1:2000)<br>goat anti-rabbit HRP (Cell Signaling 7074S, dilution 1:2000)<br>rabbit anti-mouse HRP (Dako P0260, dilution 1:2000)<br>donkey anti-rabbit (Licor 926-32213, dilution 1:10.000)<br>goat anti-mouse (Licor 925-68070, dilution 1:10.000) |
| Validation | All antibodies are well established in the lab and have been used according to the manufacturers instructions. |

## Eukaryotic cell lines

Policy information about cell lines and Sex and Gender in Research

| | |
|---|---|
| Cell line source(s) | TaC12 (bovine cell line)<br>BoMac (bovine cell line)<br>HAP1 (human cell line)<br>HEK (human cell line) |
| Authentication | None of the cell lines were authenticated. |
| Mycoplasma contamination | All cell lines were tested negative. |
| Commonly misidentified lines (See ICLAC register) | HEK |

## Animals and other research organisms

Policy information about studies involving animals; ARRIVE guidelines recommended for reporting animal research, and Sex and Gender in Research

| | |
|---|---|
| Laboratory animals | Balb/c female |
| Wild animals | - |
| Reporting on sex | Mice were only used to maintain Plasmodium sporozoites. |
| Field-collected samples | - |
| Ethics oversight | Commission for Animal Experimentation of the Cantonal Veterinary Office of Bern |

Note that full information on the approval of the study protocol must also be provided in the manuscript.

## Plants

| | |
|---|---|
| Seed stocks | *Report on the source of all seed stocks or other plant material used. If applicable, state the seed stock centre and catalogue number. If plant specimens were collected from the field, describe the collection location, date and sampling procedures.* |
| Novel plant genotypes | *Describe the methods by which all novel plant genotypes were produced. This includes those generated by transgenic approaches, gene editing, chemical/radiation-based mutagenesis and hybridization. For transgenic lines, describe the transformation method, the number of independent lines analyzed and the generation upon which experiments were performed. For gene-edited lines, describe the editor used, the endogenous sequence targeted for editing, the targeting guide RNA sequence (if applicable) and how the editor was applied.* |
| Authentication | *Describe any authentication procedures for each seed stock used or novel genotype generated. Describe any experiments used to assess the effect of a mutation and, where applicable, how potential secondary effects (e.g. second site T-DNA insertions, mosiacism, off-target gene editing) were examined.* |
